# Reducing Time to Pain Medication Administration for Pediatric Patients with Long Bone Fractures in the Emergency Department

**DOI:** 10.1097/pq9.0000000000000120

**Published:** 2018-11-15

**Authors:** Sarah S. Schuman, Rebecca B. Regen, Lindsay H. Stuart, Camden Harrell, Tamekia L. Jones, Barbara M. Stewart, Allyson M. Berg, Mindy Longjohn, Rudy J. Kink

**Affiliations:** From the * Department of Pharmacy, Le Bonheur Children’s Hospital, Department of Clinical Pharmacy and Translational Science, The University of Tennessee Health Science Center; † Children’s Foundation Research Institute, Le Bonheur Children’s Hospital; ‡ Children’s Foundation Research Institute, Le Bonheur Children’s Hospital, Department of Pediatrics, Department of Preventative Medicine, The University of Tennessee Health Science Center; § Emergency Department, Le Bonheur Children’s Hospital; ¶ Division of Pediatric Emergency Medicine, Department of Pediatric Medicine, Le Bonheur Children’s Hospital, The University of Tennessee Health Science Center.

## Abstract

**Introduction:**

Pain management is a critical aspect of effective long bone fracture treatment. Pediatric patients frequently report suboptimal pain management, which is an area of growing public concern. The purpose of this quality improvement project was to develop a protocol with the goal to administer pain medication to children presenting with suspected long bone fractures ≤47 minutes of emergency department arrival.

**Methods:**

A multidisciplinary team developed a standardized protocol for pain management of patients presenting with musculoskeletal pain utilizing acetaminophen as the first-line agent under a nurse-initiated order. Following education and implementation, weekly reports generated using the International Classification of Diseases codes of fractures were reviewed to assess compliance with the protocol. This study evaluates the frequency of a second pain medication administration and reduction in vital signs and pain scores.

**Results:**

Implementation of a pain management protocol reduced median time to pain medication administration to 26 minutes. Overall, 63% (n = 638) of patients required a second pain medication. Of these, 66.5% (348/523) who initially received acetaminophen and 59.7% (286/479) who initially received an opioid required a second pain medication. No significant changes in pre and posttreatment vital signs were found between groups. Patients who initially received opioids experienced a greater reduction in posttreatment pain scores.

**Conclusions:**

Using a standardized pain management protocol in combination with comprehensive education effectively reduces median time to pain medication administration in pediatric patients with long bone fractures. Acetaminophen is a rapid and effective first-line agent for managing pain in this population.

## INTRODUCTION

Pain management is a critical aspect of the emergency care of pediatric patients with suspected long bone fractures; however, pain is often undertreated in this patient population.^1^ Reports of suboptimal pain management in pediatric populations prompted several organizations, such as the American Pain Society and The Joint Commission, to formulate and revise protocols to ensure adequate pain management.^2^ In particular, the Center of Medicare and Medicaid Services (CMS) responded by incorporating pain management into their hospital quality standards. In 2012, CMS added the Median Time to Pain Management for Long Bone Fracture core measure to evaluate the “median time from emergency department (ED) arrival to time of initial oral, intranasal (IN), or parenteral pain medication administration for ED patients with a principal diagnosis of long bone fracture” (OP-21).^3^ Heilman et al.^4^ described a similar quality improvement process for pain medication administration utilizing nurse-initiated orders, documentation, and oral pain medication administration in triage. They reported a reduction in median time to pain medication administration from 95 minutes to 49 minutes.^4^

From June 2014 to December 2014, Le Bonheur Children’s Hospital did not effectively meet the CMS OP-21 core measure. During this period, the median time to pain medication administration from ED arrival was 71.5 minutes. The goal of a new protocol was to administer pain medication to patients with suspected long bone fractures ≤47 minutes of ED arrival through nurse-initiated medication ordering in triage. The committee chose the goal of 47 minutes because it placed the Le Bonheur ED in the top quartile benchmark when compared with similar institutions. The protocol established acetaminophen as the first-line agent based on ease of access and safety profile. Acetaminophen is an effective pain medication frequently used in pediatric EDs.^5,6^

## METHODS

### Context

Le Bonheur Children’s Hospital is a 255-bed tertiary care children’s hospital located in Memphis, Tennessee, and includes a level 1 pediatric trauma center. The 54-bed ED has an average volume of 90,000 patient visits per year. In the ED, patients are evaluated and treated by fellowship-trained pediatric emergency medicine specialists, general pediatricians, and physician trainees (fellows and residents).

### Interventions

A multidisciplinary committee comprised of ED physicians, hospitalists, ED nurses, pharmacists, information technology specialists, and hospital administrators established a protocol to address reducing administration times for pain medication. During the initial phase of the study, the committee met monthly to discuss implementation and evaluate its sustainability. Meeting frequency moved to quarterly before being dissolved. Pain medication administration times were monitored in the subsequent phase, and if the goal time were exceeded, the committee would reconvene.

The committee established the goal as a median time of ≤47 minutes from ED arrival to pain medication administration for patients who present to the ED with musculoskeletal pain. Factors that contributed most to delay in care are time to triage assessment and time to physician assessment. These factors are primarily due to the department’s high patient volume. Not all long bone fractures have an obvious deformity; therefore, the protocol encompassed all musculoskeletal pain, including but not limited to, patients who present with obvious and suspected fractures, joint dislocations, immobility, inability to bear weight, guarding of an extremity, sprains, and nonaccidental trauma. By broadening the scope of the protocol, the committee sought to limit injuries that could be under triaged initially. Based on safety profile and ease of access, acetaminophen is the first-line medication to be administered under a nurse-initiated triage order for patients 3 months and older. Several non-narcotic analgesic options are available; however, nonsteroidal anti-inflammatory drugs may pose safety concerns in patients with renal dysfunction or bleeding disorders. Acetaminophen is stored securely in ED triage rooms and medication dispensing cabinets throughout the department.

**Fig. 1.**
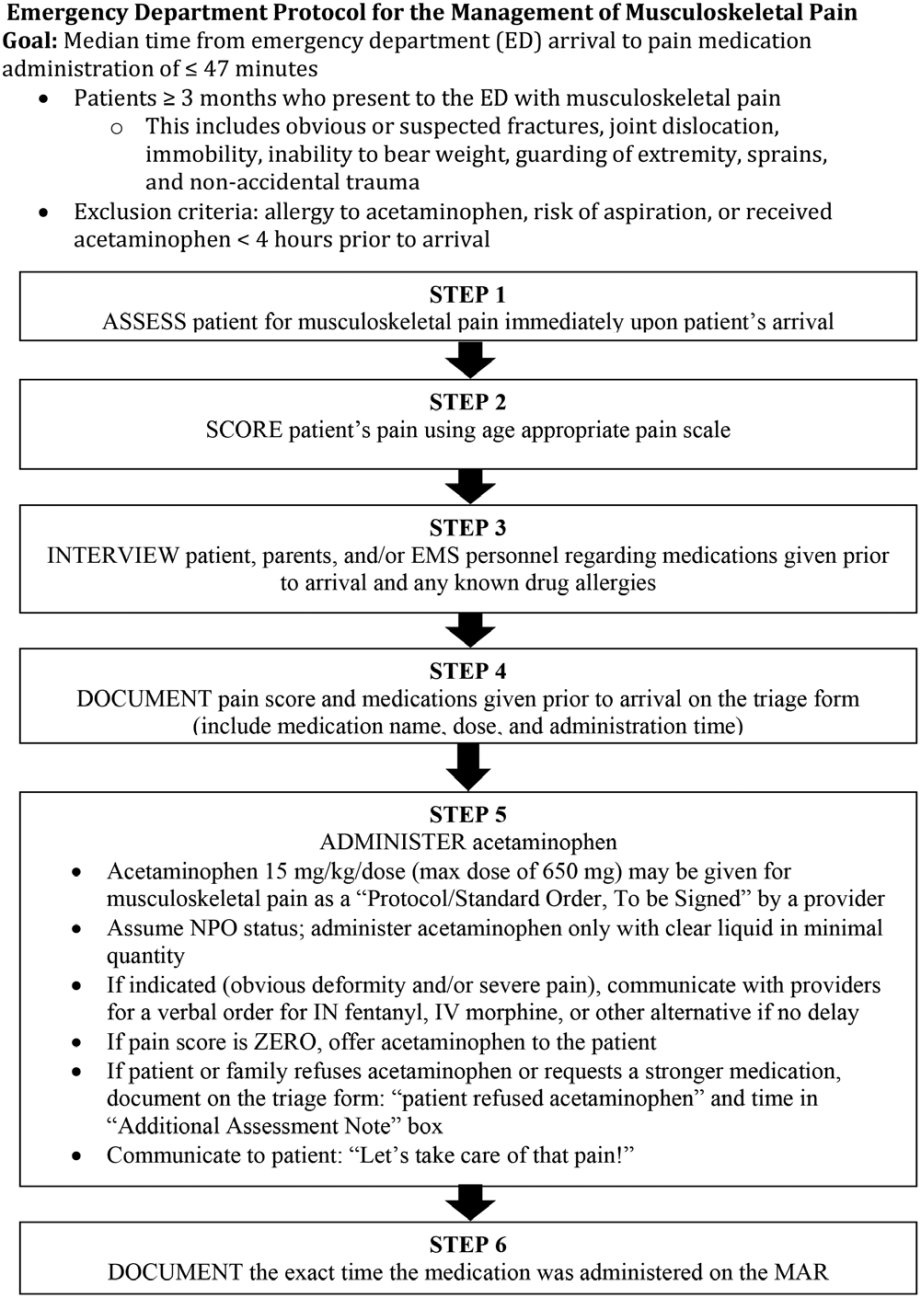
Stepwise protocol describes the clinical algorithm for managing musculoskeletal pain in pediatric patients 3 months and older presenting with suspected long bone fracture.

The stepwise protocol created a standard practice of care for the ED staff to rapidly identify patients with potential or suspected long bone fractures and initiate pain management. Implementation began in January 2015 with coinciding education for the staff. ED pharmacists and nursing supervisors educated physicians, nurses, ED technicians (EDTs), and inpatient pharmacists regarding the rationale, goals, and the protocol process. Not only is the protocol applicable to patients presenting to the waiting room but also via emergency transport vehicles; therefore, the entire team needed to understand the protocol, roles, and responsibilities in different scenarios. Committee members placed protocol flow charts (Fig. 1) and standardized dosing charts for acetaminophen in triage rooms and distributed “badge buddies” with pain scales to the staff. Other educational methods included individual or group training, online instruction, handouts, and presentations at staff meetings.

When patients present to the ED waiting room, the triage greeter (nurse or EDT) records the chief complaint on a paper assessment form. Pain evaluation was added to the paper assessment form as an additional protocol intervention. This brief evaluation included a section to document pain score, pain medication before arrival (name, dose, time), and initiation of acetaminophen order (yes or no). A patient chart is created in the electronic medical record at the registration desk using this assessment form. The triage greeter notifies a triage nurse to initiate the triage process as quickly as possible after registration if the musculoskeletal pain is reported. Patients who present via emergency transport vehicles for immediate bedding in the ED are triaged at the bedside by a nurse who completes the triage form in the electronic medical record.

The initial step of the protocol is to assess the patient for musculoskeletal pain immediately upon arrival, followed by scoring of the patient’s pain using an appropriate pain scale for the developmental stage of the child. The ED uses 3 pain scales: Face, Legs, Activity, Cry, Consolibility scale, FACES scale, and numeric pain scale (0–10).^7–9^ Generally at Le Bonheur, the ED staff utilizes Face, Legs, Activity, Cry, Consolibility scale for children 2 years and younger and the FACES scale for school-age children. The numeric pain scale is usually reserved for children 8 years and older. Next, the patient, parent, and/or emergency medical responder undergo an interview regarding medications given before arrival and any known drug allergies. The triage nurse documents the pain score and prearrival medications on the triage form in the electronic medical record. Exclusion criteria include an allergy to acetaminophen, risk of aspiration, or received acetaminophen within 4 hours before arrival. If no exclusion criteria exist, acetaminophen 15 mg/kg per dose (maximum of 650 mg) is ordered as a protocol order, to be signed electronically by an attending ED physician, then administered to the patient. Nurses administer acetaminophen as 160 mg/5 mL suspension or 325 mg tablets and document the time of administration in the patient’s chart. Nil per os status is assumed for all patients; therefore, nurses administer acetaminophen with clear liquid in minimal quantity. Nurses can request verbal medication orders from physicians, such as IN fentanyl or intravenous (IV) morphine if additional pain control is indicated as long as the administration is not significantly delayed. Once the protocol became routinely initiated, more liberty was given to the nurses to choose pain medications other than acetaminophen as the initial medication administered to patients with obvious deformities and/or patients complaining of severe pain. Alternative medications, such as acetaminophen-hydrocodone, IN/IV fentanyl, or IV morphine are available following a physician order if there is no delay.

Following implementation, ED pharmacists reviewed weekly reports generated using the International Classification of Diseases codes of long bone fractures to assess compliance. The protocol applied to patients 3 months and older who presented to the ED with musculoskeletal pain. However, this study only included patients 2 years and older with confirmed long bone fractures. The age cutoff was based on the inclusion parameter of ≥2 years used in the CMS OP-21 core measure.^3^ Exclusion from the analysis was based on the following criteria: pain medication administered before arrival, the parent or patient refused pain medication, unknown time of injury, or injury occurring ≥24 hours before presentation. The committee shared average administration times on a weekly basis with the ED team. Charts of patients who did not receive pain medications ≤47 minutes of arrival were further reviewed and evaluated by a nursing supervisor who then provided education to the department or individual as needed. Performance evaluations for nurses and EDTs included adherence to the protocol and reduction in administration times as a department goal.

### Measures

The primary outcome measure is establishing a median time from ED arrival to pain medication administration of ≤47 minutes for patients with confirmed long bone fractures. This analysis includes patients 2 years to younger than 18 years from January 2015 to October 2016 based on CMS OP-21 parameters; this core measure limits inclusion to patients older than 2 years of age. Secondary outcomes include administration of a second pain medication and reduction in vital sign values and pain scores.

Data points collected for analysis include patient demographics (age, weight, sex, race, and ethnicity), discharge diagnosis, International Classification of Diseases codes, first pain medication administered and administration time, and subsequent pain medication(s) administered and administration time(s). Vital signs pre- and postpain medication administration including heart rate, systolic blood pressure, diastolic blood pressure, and respiration rate were collected. Pre- and posttreatment pain scores were recorded. Pain scores were reported for the overall group and for the subset of subjects scored utilizing the same pain scale for both pre- and posttreatment scores.

### Analysis

All continuous variables are presented as medians and interquartile ranges (IQRs) while categorical variables are presented as frequencies and percentages. Descriptive statistics summarize all vital signs and pain scores before and within 2 hours after the administration of the medication. Patients are stratified according to pain medications received and age groups. Boxplots demonstrate a visual representation of the change between pre- and posttreatment vital signs. A line graph shows the median time to pain medication administration by month before and after the implementation of the protocol. Only the first ED visit during the study period for each patient was used in analyses. Statistical analyses were performed using SAS 9.4 (SAS Institute, Cary, N.C.). The institutional review board approved this retrospective review at the University of Tennessee Health Science Center.

## RESULTS

**Fig. 2.**
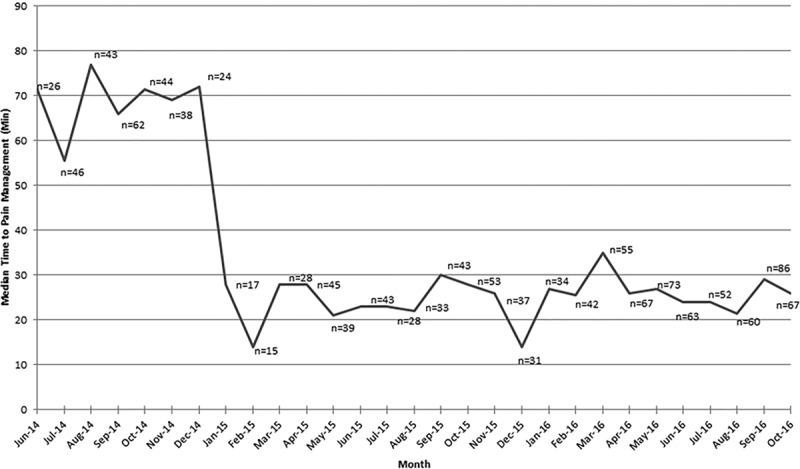
Graph depicts median time (minutes) from arrival to pain medication administration by month before the protocol implementation in January 2015 and following implementation. Number of patients included for analysis by month is shown above each time point.

There were 1,011 patients aged 2 to younger than 18 years with confirmed long bone fractures who presented to the ED during the study period. The median (IQR) age was 8.98 (5.9–12.2) years. Sixty-seven percentage of patients were male, and 53% of patients were African American. Demographics are shown in Table 1 for the entire study population and each initial pain medication. The overall median (IQR) time from ED arrival to pain medication administration was 26 (15–39) minutes. Overall, the median time to medication administration decreased from 71.5 minutes preprotocol to 26 minutes postprotocol implementation. The median time for pain medication administration was consistently below the goal of ≤47 minutes during the study period. This is shown in the line graph in Figure 2 with the median time and sample size for each month pre- and postprotocol implementation.

**Table 1.**
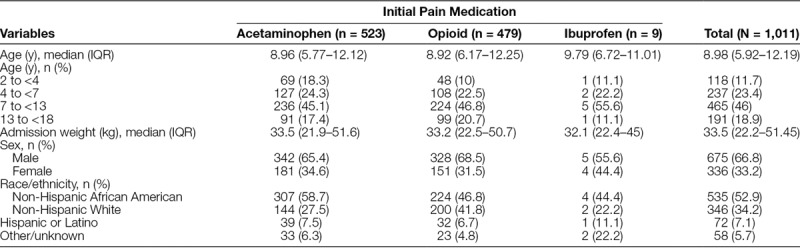
Patient Demographics

**Fig. 3.**
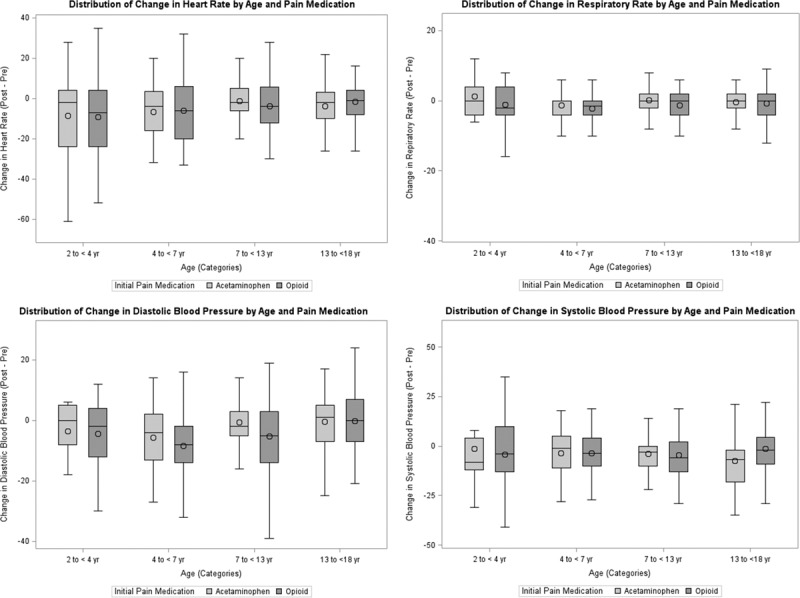
Box plots depict change in vital signs for each age group and medication. The horizontal line and circles represent the median and mean, respectively. The IQR is represented by the box. The minimum and maximum values are denoted by the whiskers.

Eighty-four percentage of patients (n = 846) received a pain medication ≤47 minutes of ED arrival with 54% of them receiving acetaminophen. For the entire study population, 52% of patients (n = 523) received acetaminophen as their initial pain medication with a median (IQR) time to administration of 20 (11–32) minutes. Patients who received an opioid as their initial medication (n = 479), including acetaminophen-hydrocodone, acetaminophen-oxycodone, fentanyl, morphine, and hydromorphone had a median (IQR) time to administration of 29 (22–47) minutes. Of these patients, 368 (76.8%) received IN fentanyl initially. Nine patients received ibuprofen as their first medication with a median administration time of 58 (27–78) minutes. Sixty-three percentage (n = 638) of patients required a second pain medication. Of these, 66.5% (348/523) who initially received acetaminophen and 59.7% (286/479) who initially received an opioid required a second medication. Changes in pre- and posttreatment vital signs are shown in Figure 3. The boxplots display the changes in vital signs for each age group and initial pain medication. Visually, the distributions of the change in vital signs were similar between the 2 medications for the corresponding age groups. Based on the results in Table 2, patients who initially received opioid medications (n = 441) had higher pretreatment pain scores with a median (IQR) score of 7 (4–10) compared with pretreatment scores of those who initially received acetaminophen (n = 494) with a median score of 5 (2–8). When comparing posttreatment scores, patients in the opioid group (n = 357) experienced a greater reduction in posttreatment pain scores with a median (IQR) score of 2 (0–6), while the acetaminophen group (n = 355) decreased to 3 (0–7). The difference in the pre- and posttreatment scores reflected a greater reduction for the opioid group (n = 325) with scores -3.5 (-5.5-(-1)) compared with 0 (-3-0) in the acetaminophen group (n = 328). These results are purely descriptive as no inference techniques were performed due to a large amount of missing data at either the pre- or posttime points.

**Table 2.**
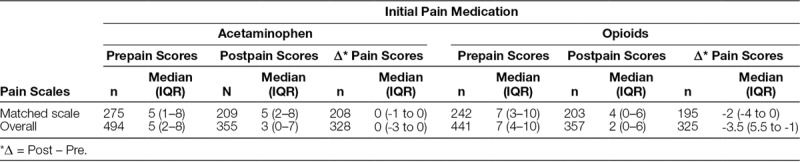
Pre- and Posttreatment Pain Scores by Scales and Medication

## DISCUSSION

Decades of literature have documented suboptimal management of pediatric pain in ED settings, prompting the World Health Organization to declare pediatric pain treatment a public health concern of major significance.^1,10^ Several barriers attribute to inadequate treatment of pediatric pain including concern of prescribing opioids to children, fear of adverse drug reactions, and difficulty assessing pain in pediatric patients.^11^ Various factors such as fear, anxiety, and lack of social support can further exacerbate pain in pediatric patients making it difficult to properly assess pain in these patients.^2^ This can lead to increased pain stimuli and psychological enhancement of pain known as hyperalgesia. Therefore, early and aggressive management of pain is recommended.^12^ Before implementation of this pain protocol, the median time from ED arrival to pain medication administration was 71.5 minutes over a 7-month period. The goal was to reduce this time to ≤47 minutes to provide the highest quality of care to patients and significantly improve quality scores, which impact reimbursement rates and top-tier designation.

The primary objective assessed the efficacy of a standardized pain management protocol in reducing median time to pain medication administration in pediatric patients with long bone fractures to ≤47 minutes. The median time was consistently ≤47 minutes during the study period, demonstrating the sustainability of the protocol. Standardizing a stepwise protocol through a multidisciplinary approach and comprehensive education for all members was critical to the success of implementation. By broadening the protocol to encompass musculoskeletal pain, potential fractures were less likely to be missed. Nurse-initiated orders facilitated faster administration of acetaminophen before physician assessment. Assessing pain in musculoskeletal injuries upon ED arrival is now second nature for the team and is emphasized upon the addition of any new team members.

Secondarily, several parameters evaluated the efficacy of acetaminophen as a first-line agent. The committee chose acetaminophen as a first-line agent due to ease of administration and proven safety profile in the pediatric population.^13^ Nonsteroidal anti-inflammatory drugs and opioids may not be appropriate for all patients. IV administration may cause delays due to order verification process and/or obtaining IV access. Additionally, national medication shortages have significantly affected the availability of many commonly used opioids limiting their use.

Use of a nonopioid analgesic, such as acetaminophen, is an effective opioid-sparing technique. Limiting opioid usage can reduce the incidence of opioid-related adverse effects such as respiratory depression, nausea, vomiting, and addiction.^2^ To ensure exceptional patient care, the protocol allows for administration of a second pain medication, including opioids, when the provider deems necessary. However, this was only needed in 66.5% of patients initially receiving acetaminophen.

Similar differences between the acetaminophen and opioid groups were found when evaluating changes in pre- and posttreatment vital signs. Vital signs are reported in age-stratified groups to represent age-dependent variability better. Patients receiving opioids had a greater reduction in pain scores across all age groups; however, patients in the opioid group had higher initial pain scores, potentially indicating more severe or painful injuries in this group. Additionally, pain scales used for assessment were inconsistent. To account for this, results for patients whose pre- and posttreatment pain scores were measured with the same scale and overall pain scores regardless of the pain scale used. During the implementation of the protocol, a task reminder was added to the electronic medical record to notify the ED team to reassess pain following the administration of a pain medication. Opioids provide stronger analgesia than non-narcotic analgesics; however, in patients with lower baseline pain, acetaminophen is likely sufficient. Of those who initially received acetaminophen, 66.5% required additional pain medication while 59.7% of those who initially received an opioid required a second medication. Given the clinical similarity in vital signs between medication groups and the comparable need for additional pain medications, acetaminophen is an acceptable first-line option in this study’s population.

There are several limitations to this study including potential lack of generalizability. Nurse-initiated medication orders and access to acetaminophen were crucial for the success of this protocol given the department’s high census; therefore, other institutions may not be able to incorporate a similar process. Inconsistent use of patient pain scales may have led to measurement bias. Another limitation was missing vital sign and pain score documentation, which eliminated several patients from certain evaluations and restricted the use of statistical inference techniques. This study did not evaluate adverse events. Finally, revisions to the protocol allow triage nurses to choose among pain medication options, which were ultimately up to provider discretion.

## CONCLUSIONS

Implementation of a standardized stepwise pain protocol resulted in a median time to pain medication administration consistently less than the goal of ≤47 minutes. Acetaminophen was effectively administered under a nurse-initiated order protocol and is an acceptable immediate first-line option for pain control in pediatric patients with suspected long bone fractures. Through the use of this protocol, the ED complies with CMS guidelines without compromising a high standard of patient care.

## DISCLOSURE

The authors have no financial interest to declare in relation to the content of this article.
